# Surveillance of antimalarial drug resistance in China in the 1980s–1990s

**DOI:** 10.1186/2049-9957-3-8

**Published:** 2014-02-24

**Authors:** De-quan Liu

**Affiliations:** 1 National Institute of Parasitic Diseases, Chinese Center for Disease Control and Prevention, 200025 Shanghai, China

**Keywords:** *Plasmodium falciparum*, Resistance, Sensitivity, Antimalarial drugs, *in vivo*, *in vitro*, Surveillance

## Abstract

Since the successful preparation of the microplates and the medium for field application, the resistance degree and its geographical distribution of chloroquine-resistant *Plasmodium falciparum*, the fluctuation of the resistance degree of *P. falciparum* to chloroquine, and the sensitivity of the parasite to commonly used antimalarial drugs were investigated between 1980 and 2003 by the *in vitro* microtest and the *in vivo* four-week test recommended by the World Health Organization (WHO). The results indicated that chloroquine-resistant falciparum malaria was present in all eight provinces/autonomous regions endemic for falciparum malaria in China, and the resistance was high and widely distributed in the Hainan and Yunnan provinces. When the use of chloroquine was stopped or administered in a decreased quanity, the drug resistance gradually decreased. In Hainan and Yunnan, *P. falciparum* was still highly resistant to chloroquine, amodiaquine and piperaquine, and sensitive to pyronaridine and artemisinin derivatives, but the sensitivity was gradually reduced. Based on these results, principles and therapeutic regimens for antimalarial drug use in China were formulated, the use of the antimalarials which had already developed resistance was stopped or reduced, and recommendations to use artemisinin derivatives or compound pyronaridine to promote a rational use of antimalarials and strengthen malaria control were made. The results showed that malaria incidence had declined, and endemic areas of falciparum malaria have been gradually reducing since the mid-1980s.

## Multilingual abstracts

Please see Additional file 1 for translations of the abstract into the six official working languages of the United Nations.

## Review

Both *P. falciparum* and *P. vivax* can develop drug resistance, but the resistance is much more important in *P. falciparum*, not only for its high rate and degree of resistance and wide geographical distribution, but also for its ability to cause high mortality. The resistance of *P. falciparum* to quinine, proguanil and cycloguanil, pyrimethamine, chloroquine was reported in the early 20^th^ century, late 1940s, mid-1950s and late 1950s respectively. By the end of the 1950s, chloroquine-resistant malaria cases were found in Colombia and Thailand. Since then, the chloroquine resistance of the parasite rapidly spread to almost all falciparum malaria endemic areas except for Central America [1], and multiple drug resistance appeared [2,3]. Presently, *P. falciparum* has developed resistance to almost all available antimalarial drugs. In recent years, *P. vivax* has also developed resistance to chloroquine [4,5]. The development of drug resistance of malaria parasites is faster than any new drug development. It has become a worldwide problem, causing more serious malaria transmission in some areas or countries, recurrence of malaria transmission in areas where malaria transmission was once interrupted, and a significant increase of morbidity and mortality, as well as noticeable increases in the cost of malaria control. Resistance of *P. falciparum* to pyrimethamine, proguanil, and cycloguanil has long been reported, but resistance to chloroquine was reported only at later stages. Chloroquine resistance of *P. falciparum* was found in the Yunnan Province [6,7] and the Hainan Province in 1973 [8,9] and 1974, respectively. Since then, it has spread widely throughout the endemic areas of falciparum malaria in China in the late 1970s to early 1980s, and either resistance to new antimalarials appeared or sensitivity to drugs reduced gradually, resulting in an increase of malaria cases and posing a major challenge for malaria control. Therefore, drug resistance of falciparum malaria became an urgent issue to tackle in malaria control in China. Before 1978, the WHO standard *in vivo* tests [10] (seven-day or four-week tests) were used for the assessment of drug resistance, but the tests are time consuming and costly, the patients need to be hospitalized (and are usually not compliant), and the result of assessment is affected by the patients’ immunity, thus it is not applicable in large areas. In 1979, an *in vitro* microtechnique was recommended by the WHO to assess drug resistance of *P. falciparum*, which was simpler, fast, accurate and acceptable, and therefore appropriate to use in a large-scale survey [11,12]. The WHO had published updated protocol for antimalarial drug resistance surveillance *in vivo* (2003), *in vitro* (2001), and genotyping method (2007) [13-15]. In 1978, the authors had successfully established *in vitro* continuous culture of erythrocytic stage *P. falciparum*, which provided an appropriate environment for the development of the *in vitro* microtechnique, including the drug-coated plates and the medium. Based on these technical progresses [16,17], a large-scale assessment of drug resistance was carried out by using both *in vivo* and *in vitro* tests between 1980 and 2003. The results of the investigation were highly beneficial to advise on using antimalarial drugs rationally in the malaria control program.

### Developing an *in vitro* microtechnique for determining the sensitivity of *P. falciparum* to antimalarial drugs

To undertake a field survey by the *in vitro* microtechnique, it is necessary to first prepare a drug-coated plate and a medium, both of which are applicable in the field. The WHO developed a standard chloroquine-coated plate and test kit in 1979 [12]. After a trial application, we found that its medium must be made into a culture solution in the field using a relatively cumbersome procedure. In addition, it is easy to pollute, its success rate is low, and the effective period is only 48 hours at 4°C. The maximum dosage of chloroquine in a chloroquine-coated plate was 32 pmol/well, which could not inhibit the growth and development of *P. falciparum* completely, and it had only one control well for observing the growth and development of the parasite. The WHO chloroquine-coated microplate had 96 wells for testing 12 cases; therefore each plate must be put into an incubator repeatedly, which may cause an error between the before and after results. In order to promote the application of the technique to better understand the situation on the drug sensitivity of *P. falciparum* in China, we started to develop drug-coated plates and field applicable mediums in 1979.

A homemade plastic plate suitable for malaria parasite growth was selected to make the chloroquine-coated microplate. Each plate had four horizontal rows with 10 wells in each row, a total of 40 wells. The size and depth of the well were the same as in the WHO plate. The chloroquine diphosphate solution was filled into the wells with reference to the dosage of the WHO chloroquine-coated microplate, two more wells were added—one for higher dosage and one for blank control [16]—which were kept in an incubator for 24 hours, the wells were sealed with plastic adhesive tape after drying, the drug-coated plate was put into a plastic bag, and stored at room temperature. Both laboratory and field tests indicated that the plate was as effective as the WHO standard plate, suitable for field use in areas with higher chloroquine resistance, and could provide a timely observation on the growth and development of malaria parasites. All these factors resulted in the test success rate increasing. When stored at room temperature for over a year, the effect remains the same [18,19].

Based on the successful preparation of the chloroquine-coated plate, other antimalarial drug-coated plates were also developed, including piperaquine, pyronaridine, artesunate, dihydroartemisinin, artemether, and arteether. Consequently, we could make simultaneous determinations of the sensitivity of one patient’s blood to different antimalarials, and overcome the adverse effects on the growth of malaria parasites due to repeatedly entering the incubator and sealing with plastic tape. At present, the *in vitro* microtechnique can be used to determine the sensitivity of the malaria parasite to all commonly used antimalarials.

Based on the experience with the *in vitro* continuous culture of *P. falciparum*, and by carrying out repeated tests and improvements, the liquid medium was prepared with RPMI1640 powder, adding 15% type AB human serum, then packed into sterile ampoules with 0.9 ml of the solution in each; then after lyophilization, the ampoule was sealed and stored at 4°C. Before use, it was dissolved with 0.21% sodium bicarbonate solution (in ampoule). This medium was plainly packed, easily prepared, and used in field situations; one ampoule of medium for one sample. Comparative tests demonstrated that the above homemade lyophilized medium was better than the WHO standard medium in supporting the growth of malaria parasites, and the effect was kept stable for two years at 4°C [16,18,19].

Since the preparation of lyophilized medium was still troublesome, ampoule-sealed liquid medium was prepared later. Once it was opened, it could be immediately used for testing, was much easier to apply in the field, and could remain in its effective state for two months at 4°C. It was well accepted by various institutions in the country together with the drug-coated plates [17].

### The chloroquine-resistance degree of *P. falciparum* and its geographical distribution

A large-scale survey on the chloroquine resistance of *P. falciparum* was then carried out in the 1980s in eight provinces/autonomous regions where falciparum malaria was prevalent, namely Yunnan, Hainan, Guangxi, Guizhou, Henan, Jiangsu, Anhui, and Fujian. Based on the local endemicity of falciparum malaria, each province/autonomous region set pilot points in counties with high morbidity of falciparum malaria to enroll patients, and to make an *in vivo* four-week test [10] and an *in vitro* microtest [11,12]. Between 1981 and 1984, 466 cases from 23 counties were examined. Among them, 395 cases finished the treatment course and received observation four weeks later; 311 out of 321 cases had successful *in vitro*, and 224 cases were examined concurrently by *in vivo* and *in vitro* with success. The results indicated that chloroquine resistance had already existed in the eight provinces/autonomous regions, especially in Hainan and Yunnan where falciparum malaria was severely prevalent. In Hainan, 106 cases from four counties were examined by the *in vivo* test; 90 cases were successful, 74 were chloroquine resistant (82.2%), and 35.1% had degree III resistance (RIII). Meanwhile, 123 cases from six counties were examined by the *in vitro* microtechnique; 120 cases were successful (86 cases were concurrently examined by the *in vivo* test), and 113 were chloroquine resistant (94.2%), which was more severe in the southwestern mountainous area [8,9,20]. In Yunnan, 178 cases from four counties were examined by the *in vivo* four-week test; 155 were successful and 115 (74.2%) had chloroquine resistance. Among 93 cases from four counties examined by the *in vitro* microtest, 88 cases were successful (81 cased were also examined by the *in vivo* test), 75 cases (85.2%) were chloroquine resistant, and the resistance was severe in the bordering area of southern Yunnan [6,7]. In Guangxi, of the 46 cases examined by the *in vivo* test, 36 were successful and only 12 cases (33.3%) were RI; 14 of the successful cases were sensitive. Among 28 cases examined by the *in vitro* microtest, 27 were successful and 21 (77.8%) were chloroquine resistant. In Guizhou, 39 cases were examined by the *in vivo* test; 31 were successful, two (6.5%) were RI. Among the 34 examined by the *in vitro* microtest, 33 were successful and 12 cases (36.4%) appeared to show resistance. In Anhui, of the 25 cases examined by the *in vivo* four-week test, 20 were successful, four were RI, and 16 were RII. All 22 cases examined by the *in vitro* microtest developed resistance. In Henan, of the 62 cases examined by the *in vivo* four-week test, 52 were successful and three were RI. Of the nine cases examined by the *in vitro* test, three (33.3%) were chloroquine resistant. In Jiangsu, eight cases examined by the *in vivo* four-week test were sensitive to chloroquine, but five out of 12 cases (41.7%) examined by the *in vitro* microtest showed resistance. In Fujian, one of the two cases examined by the *in vivo* four-week test was RI. The results indicated that falciparum malaria highly resistant to chloroquine was present in Hainan and Yunnan provinces, and falciarum malaria in southern Guangxi and central Anhui also exhibited obvious chloroquine resistance, but the resistance level in these latter two provinces was lower than that in Hainan and Yunnan, and the chloroquine resistance in southern Henan, Guizhou, and western Jiangsu was at its initial stage [21] (see Table 1).

**Table 1:** Result of the assessment of the chloroquine resistance of P. falciparum

***Province***	***In Vivo test***			***In Vitro test***		
	***No. cases examined***	***No. resistant cases***	***Resistance rate *** **(%)**	***No. cases examined***	***No. resistant cases***	***Resistance rate *** **(%)**
Hainan	90	74	82.2	120	113	94.2
Yunnan	155	115	74.2	88	75	85.2
Guangxi	37	12	32.4	27	21	77.8
Guizhou	31	2	6.5	33	12	36.4
Anhui	20	20	100	22	22	100
Henan	52	3	5.8	9	3	33.3
Jiangsu	8	0	0	12	5	41.7
Fujian	2	1	50.0			

The above results indicated the corelationship between *in vivo* and *in vitro* tests. All 135 resistant cases determined by the *in vivo* test were also resistant by the *in vitro* test; and all 40 sensitive cases determined by the *in vitro* test also proved to be sensitive by the *in vivo* test. However, among the 95 sensitive cases determined by the *in vivo* test, 55 cases showed resistance at different levels by the *in vitro* test, indicating that some cases could not be detected by the *in vivo* test; thereby, the resistance rate by the *in vitro* test would often be higher than that by the *in vivo* test. At the same time, it could be seen that the higher the resistance rate by the *in vivo* test, the higher the drug concentrations for complete inhibition of schizont maturation by the *in vitro* test [16].

### Investigation on the fluctuation of the chloroquine resistance of *P. falciparum*

Since chloroquine-resistant falciparum malaria was found in Yunnan and Hainan in 1973 and 1974, respectively, the resistant falciparum malaria began spreading rapidly, and the degree of resistance kept rising, becoming widely distributed from early individual resistant cases in just five years. In 1978, chloroquine-resistant *P. falciparum* was found in all 11 counties endemic for falciparum malaria in Hainan. Therefore, the Hainan provincial government issued a document to stop the use of chloroquine for malaria control from 1979 and replace it with piperaquine. In 1983, it was confirmed that chloroquine-resistant falciparum malaria spread all over the falciparum malaria endemic areas in Yunnan. Since then, chloroquine was rarely used, and the drugs mainly used for the control of falciparum malaria were artemisinins, pyronaridine, and the No. 3 antimalarial tablet (piperaquine + sulfadoxine). In order to understand the fluctuation of the chloroquine resistance of *P. falciparum* after stopping or reducing the use of chloroquine, the sensitivity of *P. falciparum* to chloroquine was investigated at one to three year intervals in the Hainan and Yunnan provinces.

### The results of the assessment in Hainan

*Variations in the chloroquine*-*resistance rate*: A total of 287 cases from the Baoyou Town of Ledong County were examined eight times successively by the *in vitro* microtest; among them 128 cases were concurrently examined by the *in vivo* test. The resistance rate decreased from 97.7% in 1981 to 26.7% in 1997. Taking the 1981 rate as 100, this was a reduction of 72.7% (*P* < 0.01). A total of 128 cases were examined by the *in vivo* four-week test; the resistance rate decreased from 84.2% in 1981 to 18.4% in 1997, a reduction of 78.1% (*P* < 0.01). In 2001, 82 cases from the Yaliang Township of Sanya City were examined by the *in vitro* test; the resistance rate was 59.8%. In 2003, 16 cases from the Fubao Township of Ledong County were examined by the *in vivo* four-week test; the resistance rate was 62.5%, indicating that after stopping the use of chloroquine, the chloroquine resistance of *P. falciparum* followed a downward trend.

*Variations in the chloroquine*-*resistance degree*: The results by the *in vitro* microtest demonstrated that the mean drug concentration for complete inhibition of schizont maturation decreased from 10.4 ± 7.1 pmol/μl blood in 1981 to 1.6 ± 1.5 pmol/μl blood in 1997, a reduction of 84.4% (*P* < 0.01). This indicated that in most cases, as the time of stopping the use of chloroquine extended, the necessary drug concentration for complete inhibition of schizont maturation decreased gradually from the previous higher drug concentration (>6.4 pmol/μl) to a lower drug concentration (<1.6 pmol/μl). During 1981–1997, the former decreased from 83.3% to 6.7%, a reduction of 92.0% (*P* < 0.01), and the latter increased from 4.2% to 73.3%, an increase of 94.3% (*P* < 0.01). The result of the assessment by the *in vivo* four-week test demonstrated that the asexual parasite clearance time in the blood reduced from 72 hours in 1981 to 50.7 hours in 1997, the percentage of RIII cases in the whole spectrum of resistant cases reduced from 53.1% in 1981 to 14.3% (*P* < 0.01) in 1997. In 2001, 82 cases from the Yaliang Township of Sanya City were examined by the *in vitro* microtest; the results indicated that the mean drug concentration for complete inhibition of schizont maturation was 3.56 pmol/μl blood and in 12.5% cases, it was >6.4 pmol/μl blood. In 2003, 16 cases from the Fubao Township of Ledong County were examined by the *in vivo* four-week test; the results indicated that the asexual parasite clearance time in the blood was an average of 56.9 hours and the percentage of RIII cases was 20%, indicating that the degree of the chloroquine resistance of *P. falciparum* reduced gradually after the use of chloroquine was stopped [22-26].

### The results of the assessment in Yunnan

*Variations in the chloroquine*-*resistance rate*: A total of 234 cases from the Mengla County were examined seven times successively by the *in vitro* microtest; the resistance rate decreased from 97.4% in 1981 to 77.8% in 1999, a reduction of 20.1% (*P* < 0.01). A total of 27 cases from the Jinghong County were examined by the *in vitro* microtest; the resistance rate was 70.4%.

*Variations in the chloroquine*-*resistance degree*: The mean drug concentration for complete inhibition of schizont maturation decreased from 17.2 ± 12.6 pmol/μl blood in 1981 to 4.4 ± 3.1 pmol/μl blood in 1999, a reduction of 74.4% (*P* < 0.01). The percentage of the cases with a complete inhibition of schizont maturation in the drug concentration >6.4 pmol/μl in blood decreased from 58.9% in 1981 to 19.6% in 1999, a reduction of 66.7% (*P* < 0.01). In 2002, 27 cases from the Jinghong County were examined; the mean drug concentration for complete inhibition of schizont maturation was 4.0 ± 3.3 pmol/μl blood, and 16.6% cases had complete inhibition of schizont maturation in drug concentration >6.4 pmol/μl in blood, indicating that the chloroquine-resistance rate and degree of *P. falciparum* fell gradually after reduced use of chloroquine [26] (see Table 2).

**Table 2:** Variations in the chloroquine resistance of P. falciparum (In vitro)

***Province***	***Year***	***No. of cases examined***	***Resistance rate *** **(%)**	***Mean inhibition concentration *** **(*pmol*/μ** *l*)*	**> *6.4 pmol*/μ** ***l*** ** (%)
Hainan	1981	48	97.7	10.46 ± 7.14	83.3
	1997	45	26.7	1.63 ± 1.17	6.7
Yunnan	1981	39	97.4	17.2 ± 12.6	58.9
	1999	18	77.8	4.4 ± 3.1	19.5

*The mean drug concentration for complete inhibition of schizont maturation.

**Percentage of cases with complete inhibition of schizont maturation.

### Present situation of the sensitivity of *P. falciparum* to antimalarial drugs

Due to the widespread chloroquine resistance of *P. falciparum*, application of other antimalarial drugs increased from 1980. In order to understand the sensitivity of the parasite to commonly used antimalarials and to guide rational administration of the drugs, the sensitivity of *P. falciparum* to chloroquine, amodiaquine, piperaquine, mefloquine, pyronaridine, artesunate, arteether, dihydroartemisinin, and quinine was examined three and five times in Hainan and Yunnan, respectively, between 1984 and 2002. The results indicated that the *P. falciparum* in the two provinces had developed resistance to seven antimalarial drugs except for mefloquine and quinine, and displayed relatively higher resistance to chloroquine, amodiaquine, and piperaquine. However, the sensitivity of *P. falciparum* to chloroquine was recovering. The rate and degree of resistance to piperaquine were on the rise. In the Hainan Province, a total of 216 cases were examined by the *in vitro* test five times successively; the resistance rate increased from 15.8% in 1985 to 72.9% in 1997, and the mean drug concentration for complete inhibition of schizont maturation increased from 9.7 pmol/μ in 1985 to 47.9 pmol/μl in 1997, which was almost five times higher than it was in 1985. A total of 154 cases were examined by the *in vivo* test three times succesively; the resistance rate increased from 17.2% in 1984 to 50.0% in 1997. No RIII cases were found in 1984, but RIII cases accounted for 71.4% of the total resistant cases in 1997. In the Yunnan Province, 126 cases were examined by the *in vitro* test three times successively; the resistance rate increased from 21.3% in 1990 to 73.0% in 1993, and the mean drug concentration for complete inhibition of schizont maturation increased from 19.0 pmol/μl in 1990 to 38.0 pmol/μl in 1993, which was two to three times higher than in 1990. The results of clinical treatment indicated that 50% of falciparum malaria cases in Hainan developed resistance to piperaquine (see Table 3), and the sensitivity to pyronaridine and artemisinin derivatives was falling gradually. Even artemether-resistant cases were found through clinical treatment in Yunnan [27-32]. In Hainan, the mean concentration of pyronaridine and artesunate for complete inhibition of schizont maturation *in vitro* increased by four to eight times and two to eight times, respectively [33-37] (see Table 4).

**Table 3:** Sensitivity of P. falciparum to piperaquine

***Province***	***In vitro test***				***In vivo test***			
	***Year***	***No. of cases examined***	***Resistance rate *** **(%)**	***Inhibition concentration *** **(*pmol*/μ** *l*)*	***Year***	***No. of cases examined***	***Resistance rate *** **(%)**	***RIII *** (%)**
Hainan	1985	38	15.5	9.7 ± 9.1	1984	64	17.2	0
	1997	70	72.9	47.9 ± 36.5	1997	42	50.0	71.4
Yunnan	1990	47	21.3	19.0 ± 17.2				
	1993	37	73.0	38.0 ± 32.5				

*The mean concentration of piperaquine for complete inhibition of schizont maturation.

**Percentage of RIII cases in total resistant cases.

**Table 4:** Sensitivity of P. falciparum to pyronaridine and artesunate

***Drug***	***Province***	***In vitro test***			***In vivo test***			
		***Year***	***No. of cases examined***	***Inhibition concentration *** **(*pmol*/μ** *l*)*	***Year***	***No. of cases examined***	***Defervescence time *** **(*h*)**	***Parasite clearance time *** **(*h*)**
Pyronaridine	Hainan	1986	20	0.08	1986	42	32.9 ± 12.4	43.3 ± 23.3
		1997	37	1.36	1996	13	40.2 ± 15.7	62.5 ± 28.7
	Yunnan	1988	25	0.14				
Artesunate	Hainan	1986	18	0.028				
		2002	32	0.135				
	Yunnan	1988	24	0.038				
		2002	22	0.054				

*The mean drug concentration for complete inhibition of schizont maturation.

### Rational use of antimalarial drugs in China

After completion of the survey on the resistance degree of chloroquine-resistant falciparum malaria and its geographical distribution, the results and suggestions for improving local malaria control were reported to the Ministry of Health and the local governments, recommening that surveillance on drug-resistant malaria be listed as one of the important tasks in malaria control. For those provinces/autonomous regions only with sporadic falciparum malaria cases, it was suggested that mosquito control measures were strengthened to prevent possible focal transmission of falciparum malaria, focusing more on migrants to prevent the spreading of the drug resistance more effectively. In Hainan and Yunnan, where critical drug resistance persisted, use of chloroquine for falciparum malaria control should be stopped, and the therapeutic effect of other substitutes should be monitored, in order to find and contain the new development of drug resistance in a timely fashion. Due to active implementation of the control program, the number of falciparum malaria endemic provinces/autonomous regions decreased yearly, and after 1998, falciparum malaria was prevalent only in the Hainan and Yunnan provinces.

In order to more effectively cure malaria patients and avoid or delay the development of drug resistance of *P. falciparum*, principles and therapeutic regimens for the application of antimalarial drugs in China were formulated in 2000 on the basis of the drug sensitivity of the parasite found through the surveys. They were divided into first-line, second-line, and third-line drugs, in order to make a reasonable and standardized application of the drugs. Chloroquine and piperaquine were recommended as the first-line drugs for endemic areas of vivax malaria and some areas of falciparum malaria where chloroquine and piperaquine were still sensitive. Artemisinin derivatives and pyronaridine were recommended as the second-line drugs for areas of falciparum malaria with moderate or high resistance to chloroquine and piperaquine. Artemisinin derivatives or pyronaridine in combination with other antimalarials were recommended as the third-line drugs for those areas where the therapeutic effect of second-line drugs was limited [38-46]. Considering that *P. falciparum* in China has generally developed resistance to chloroquine, piperaquine and sulfadoxine-pyrimethamine, and other antimalarials, artemisinin derivatives and pyronaridine have been widely used as the first-line drugs in endemic areas of falciparum malaria. Due to the rational and standardized use of antimalarials, the therapeutic effect was greatly improved, malaria cases decreased considerably from 903,802 cases in 1984 to 7,855 cases in 2010 (including 1,258 falciparum malaria cases), and the number of falciparum malaria endemic counties decreased from 74 in eight provinces in 1984 to just 17 in the Yunnan Province only in 2010 [47,48].

## Conclusion

Drug resistance of *P. falciparum* is a global problem, especialy in Southeast Asia. In some neighboring countries, such as Thailand, Cambodia, Myanmar, India, and Vietnam, *P. falciparum* has developed resistance to chloroquine, sulfadoxine-pyrimethamine, mefloquine, etc., extensively. On the Thailand-Cambodia border and in Myanmar, high multiple resistance has appeared [40,49], and Myanmar and Thailand have reported that *P. vivax* has developed resistance to chloroquine and primaquine, respectively [4,5]. Therefore, antimalarials have been devided into first-line drugs and second-line drugs for rational application in these countries [40]. Chloroquine, piperaquine, pyronaridine, and artemisinin derivatives are commonly used antimalarials in China. Chloroquine has been used extensively for more than 50 years, and the others are relatively new antimalarials and have been used for over 20 years.

The results of the survey indicated that there was a certain variability of the resistance of *P. falciparum* to chloroquine—the resistance rate and degree to chloroquine was increasing from the appearance of chloroquine-resistant falciparum malaria cases to the early stage of reducing or stopping the use of chloroquine. Investigations indicated that once *P. falciparum* developed resistance to chloroquine, when mosquito vectors with strong transmissibility existed (such as *Anopheles dirus* and *An. minimus*), and with frequent human population movement, falciparum malaria would spread rapidly. Once resistance to chloroquine appeared, if the use of chloroquine could be stopped in a timely manner, the parasite could gradually restore its sensitivity to chloroquine under the condition of no drug pressure, but the recovery progress would be slow. In the Hainan Province, 18 years after stopping the use of chloroquine, about 20–60% of the cases still had certain resistance to chloroquine. The process of recovery of chloroquine sensitivity firstly indicated a decline of the resistance degree, then the reduction of the resistance rate when the resistance level dropped below a certain level. In the first 10 years after stopping use of chloroquine, the resistance degree fell faster, yet the resistance rate hardly changed; however, 10 more years later, the resistance rate fell faster as well.

In the period of extensive use of piperaquine, pyronaridine, artemisinin derivatives, or the No. 3 antimalarial tablet, there were plenty of cases taking insufficient dosages due to low compliance, which was also one of the major reasons for a decrease in the sensitivity to those drugs. It was not recommended to use artemisinin derivatives and pyronaridine in endemic areas of vivax malaria; neither for treatment, preventive medicine, or presumptive treatment for individual cases. They were always sensitive and effective clinically, yet the sensitivity of *P. falciparum* to the drugs was in a gradual downward trend. In order to delay the development of resistance and protect antimalarial drugs, based on the WHO advocation to use combination artemisinin drugs, it was suggested not to use artemisinin derivatives alone, but in combination with other antimalarials [40,50,51].

## Competing interests

The author declares that they have no competing interests.

## Supplementary Material

Additional file 1 Multilingual abstracts in the six official working languages of the United Nations. Click here for file
