# Supplementary material for: Surveillance of antimalarial drug resistance in China in the 1980s–1990s
Source: Infect Dis Poverty. 2014 Feb 24;3:8. doi: 10.1186/2049-9957-3-8 (PMC3946046; doi:10.1186/2049-9957-3-8)
Supplement: Additional file 1 — Multilingual abstracts in the six official working languages of the United Nations. [file 2049-9957-3-8-S1.pdf]

Translation of the abstract into the six official working languages of the United Nations

مراقبة مقاومة العقار المضاد لمرض الملاريا في الصين في الثمانينات والتسعينات من القرن العشرين

لي ودي- كيوان

ملخص

منذ الإعداد الناجح لصفائح المعايرة ووسط التطبيق الميداني، فإن درجة المقاومة وتوزيعها الجغرافي للمتصورة المنجلية المقاومة للكلوروكين وتقلب درجة المقاومة للمتصورة المنجلية للكلوروكين وحساسية الطفيلي للعقاقير المضادة لمرض الملاريا شائعة الاستخدام تم التحقق منها ما بين عامي 1980 و2003 بواسطة الاختبارات المصغرة في المختبر واختبار الأسابيع الأربعة في المختبر الموصى بها من قِبَل منظمة الصحة العالمية (WHO).وأشارت النتائج إلى تواجد الملاريا المنجلية المقاومة للكلوروكين في جميع المقاطعات الثمانية/ ومناطق الحكم الذاتي المصابة التي يستوطن بها مرض الملاريا المنجلية في الصين، وأن المقاومة كانت مرتفعة وواسعة الانتشار في مقاطعات هايان ويونان. عند توقف استخدام الكلوروكين أو إعطاءه بكمية منخفضة، انخفضت مقاومة العقار بصورة تدريجية. كانت المتصورة المنجلية في مقاطعتي هايان ويونان مازالت مرتفعة المقاومة للكلوروكين وأمودياكين وبيبيراكين وكانت الحساسية تجاه مشتقات بيرونباردين وأرتيميسين ، ولكن الحساسية انخفضت بصورة تدريجية.

وبناء على تلك النتائج، تم وضع المبادئ والنظريات العلاجية لاستخدام العقار المضاد لمرض الملاريا في الصين وتم وقف أو تقليل استخدام العقاقير المقاومة لمرض الملاريا التي أثبتت مقاومة بالفعل، وتم وضع التوصيات باستخدام مشتقات أرتيميسين أو مركب بيرونباردين وتقوية التحكم في مرض الملاريا. أظهرت النتائج أن وقوع مرض الملاريا قد انخفض، وأن المناطق التي يستوطن بها مرض الملاريا المنجلية قد أخفضت بصورة تدريجية منذ منتصف ثمانينات القرن العشرين.

Translated from English version into Arabic by Claudia Guiraldes, through

TRANSLATORS
WITHOUT BORDERS

1980年代～1990年代中国抗疟药抗性的监测情况

刘德全

摘要

研制成功涂药板和便于现场使用的培养基后，20世纪80年代到90年代，采用体内四周法和体外微量法开展了我国抗氯喹恶性疟的抗性程度及地理分布；停用或减少使用氯喹防治恶性疟后，恶性疟原虫对氯喹抗性的消长及我国恶性疟原虫对常用抗疟药敏感性等调查。结果显示，有恶性疟流行的八省（区）均有抗氯喹恶性疟存在，其中海南和云南两省尤为严重，抗性程度高，分布地区广。停用或减少使用氯喹后，恶性疟原虫对氯喹抗性逐渐降低。海南和云南两省恶性疟原虫对氯喹、氨酚喹和哌喹有高度抗性，对咯萘啶和青蒿素类药物敏感，但敏感性在逐渐降低。根据调查结果，制定了抗疟药使用原则和用药方案，停止或减少使用已有抗性的抗疟药，推荐使用青蒿素类药物和咯萘啶及其复方，使抗疟药能合理规范使用，促进了疟疾防治，疟疾发病逐年降低，恶性疟流行区逐渐缩小。

Translated from English version into Chinese by Liu De-quan

Surveillance de la résistance aux antipaludéens en Chine dans les années 1980 et 1990

Liu De-quan

Résumé

Depuis le succès de la préparation des microlames et la possibilité de les utiliser sur le terrain, le degré de résistance et la répartition géographique du *Plasmodium falciparum* résistant à la chloroquine, la fluctuation du degré de résistance du *P. falciparum* à la chloroquine et la sensibilité du parasite aux antipaludéens couramment utilisés ont fait l'objet d'une enquête. Cette enquête s'est déroulée entre 1980 et 2003 et a été menée au moyen de microtests in vitro et des tests in vivo de quatre semaines recommandés par l'Organisation mondiale de la santé (OMS). Les résultats ont indiqué que le paludisme à falciparum résistant à la chloroquine était présent dans les huit provinces/régions chinoises autonomes dans lesquelles on constate une endémie de paludisme à falciparum et cette résistance était forte et largement distribuée dans les provinces de Hainan et Yunnan. La résistance aux médicaments a diminué progressivement après l'ârrêt ou la réduction de l'utilisation de la chloroquine. Dans la province de Hainan et Yunnan, le *P* . *falciparum* était encore très résistant à la chloroquine, à l'amodiaquine et à la piperaquine. Il restait sensible à la pyronaridine et aux dérivés de l'artémisinine, mais sa sensibilité se réduisait progressivement. En se basant sur ces résultats, des principes et schémas thérapeutiques ont été formulés pour les médicaments antipaludéens utilisés en Chine. Les médicaments antipaludéens pour lesquels une résistance avait déjà été développée n'ont plus été utilisés ou ont été moins utilisés et des recommandations ont été émises incitant à leurs préférer des dérivés de l'artémisinine ou des composés de pyronaridine afin de promouvoir une utilisation rationnelle des médicaments antipaludéens et d'intensifier la lutte contre le paludisme. Les résultats ont montré une diminution de l'incidence du paludisme et une réduction progressive des zones d'endémie de paludisme à falciparum depuis le milieu des années 1980.

Translated from English version into French by Carine Toucand, through

TRANSLATORS
WITHOUT BORDERS

Исследование устойчивости к антималярийным препаратам в Китае в 1980-х — 1990-х гг.

Лиу Де-куань

Резюме

После успешного изготовления микропланшетов и вещества для применения в полевых условиях, между 1980 и 2003 гг. было произведено исследование масштабов сопротивляемости антималярийным препаратам, географического распределения хлорохиноустойчивой формы *Plasmodium falciparum*, флуктуации степени сопротивляемости *P. falciparum* хлорохину и чувствительности паразита к обычно используемым лекарствам против малярии. Исследование включало в себя лабораторное микроиспытание и четырехнедельное испытание в полевых условиях, рекомендованное Всемирной Организацией Здравоохранения (ВОЗ). Результаты показали, что хлорохиноустойчивая тропическая малярия встречалась во всех восьми провинциях/автономных регионах Китая, эндемичных для тропической малярии, а также продемонстрировали высокий уровень сопротивляемости хлорохину и ее широкое распространение в провинциях Хайнань и Юннань. После прекращения использования хлорохина или уменьшения его дозы сопротивляемость медикаментам постепенно уменьшалась. В провинциях Хайнань и Юннань *P. falciparum* по-прежнему обладал высокой устойчивостью к хлорохину, амодиахину и пипераквину и был чувствителен к производным пиронаридина и артемизинина, но эта чувствительность постепенно снижалась. На основе этих результатов были сформулированы принципы и схемы применения антималярийных средств в Китае, использование антималярийных средств, к которым уже развилась устойчивость, было прекращено или сокращено, а также были сделаны рекомендации по использованию производных артеминизина или соединений с пиронаридином, чтобы способствовать более рациональному использованию антималярийных препаратов и усилению контроля над малярией. Результаты показали, что число случаев заболеваемости малярией уменьшилось, а площадь областей, эндемичных для тропической малярии, постепенно сокращается с середины 1980-х гг

Translated from English version into Russian by Mayya Shlyakhter, through

TRANSLATORS
WITHOUT BORDERS

Vigilancia de la resistencia de los medicamentos antimaláricos en China en las décadas de 1980 y 1990

Liu De-quan

Resumen

Desde la exitosa preparación de las microplacas y el medio para aplicación en terreno, entre 1980 y 2003 se investigó el grado de resistencia y la distribución geográfica del *Plasmodium falciparum* resistente a la cloroquina, la fluctuación del grado de resistencia del *P. falciparum* a la cloroquina y la sensibilidad del parásito a medicamentos antimaláricos utilizados comúnmente, utilizando la micro prueba *in vitro* y la prueba de cuatro semanas *in vivo* recomendadas por la Organización Mundial de la Salud (OMS). Los resultados indicaron que la malaria falcípara resistente a la cloroquina estaba presente en las ocho provincias/regiones autónomas endémicas para malaria falcípara en China y la resistencia era alta y extensamente distribuida en las provincias de Hainan y Yunnan. Cuando se dejó de utilizar cloroquina o se utilizó en cantidades reducidas, la resistencia al medicamento disminuyó paulatinamente. En Hainan y Yunnan, *P. falciparum* aún era altamente resistente a la cloroquina, amodiaquina y piperaquina y sensible a pironaridina y derivados de la artemisinina, pero la sensibilidad se redujo paulatinamente. Basándose en estos resultados, se formularon principios y pautas de tratamiento para los medicamentos antimaláricos utilizados en China, el uso de antimaláricos a los que ya se había desarrollado resistencia, se suspendió o se redujo y se efectuaron recomendaciones para la utilización de derivados de la artemisinina o compuesto de pironaridina para promover el uso racional de antimaláricos y reforzar el control de la malaria. Los resultados demostraron que la incidencia de la malaria había disminuido y que las áreas donde la malaria falcípara era endémica, se han ido reduciendo paulatinamente a partir de mediados de los años 80.

Translated from English version into Spanish by Claudia Guiraldes, through

TRANSLATORS WITHOUT BORDERS
